# Environmental Surveillance and Characterization of Antibiotic Resistant *Staphylococcus aureus* at Coastal Beaches and Rivers on the Island of Hawaiʻi

**DOI:** 10.3390/antibiotics10080980

**Published:** 2021-08-13

**Authors:** Tyler J. Gerken, Marilyn C. Roberts, Philip Dykema, Geoff Melly, Darren Lucas, Vanessa De Los Santos, Joenice Gonzalez, Patrick Butaye, Tracy N. Wiegner

**Affiliations:** 1 Department of Environmental and Occupational Health Sciences, School of Public Health, University of Washington, Seattle, WA 98105, USA; tylergerken808@gmail.com; 2 Washington State Department of Health, Shoreline, WA 98155, USA; Philip.Dykema@doh.wa.gov (P.D.); geoff.melly@doh.wa.gov (G.M.); Darren.Lucas@doh.wa.gov (D.L.); Vanessa.De_Los_Santos@doh.wa.gov (V.D.L.S.); joenice.gonzalezdeleon@doh.wa.gov (J.G.); 3 Ross University School of Veterinary Medicine, Basseterre 42123, Saint Kitts and Nevis; Pbutaye@rossvet.edu.kn; 4 Department of Pathology, Bacteriology and Avian Diseases, Faculty of Veterinary Medicine, Ghent University, 9820 Merelbeke, Belgium; 5 Marine Science Department, University of Hawaiʻi at Hilo, Hilo, HI 96720, USA; wiegner@hawaii.edu

**Keywords:** *Staphylococcus aureus*, MRSA, MSSA, whole-genome sequencing, USA300, sequence types, antimicrobial resistance, virulence factors, beaches, rivers

## Abstract

*Staphylococcus aureus* are human facultative pathogenic bacteria and can be found as contaminants in the environment. The aim of our study was to determine whether methicillin-resistant *Staphylococcus aureus* (MRSA) and methicillin-susceptible *S. aureus* (MSSA) isolated from coastal beach and river waters, anchialine pools, sand, and wastewater on the island of Hawaiʻi, Hawaiʻi, are a potential health risk. Samples were collected from three regions on Hawaiʻi Island from July to December 2020 during the COVID-19 pandemic and were characterized using whole-genome sequencing (WGS). From WGS data, multilocus sequence typing (MLST), SCC*mec* type, antimicrobial resistance genes, virulence factors, and plasmids were identified. Of the 361 samples, 98.1% were positive for *Staphylococcus* spp. and 7.2% were *S. aureus* positive (n = 26); nine MRSA and 27 MSSA strains were characterized; multiple isolates were chosen from the same sample in two sand and seven coastal beach water samples. The nine MRSA isolates were multi-drug resistant (6–9 genes) sequence type (ST) 8, clonal complex (CC) 8, SCC*mec* type IVa (USA300 clone), and were clonally related (0–16 SNP differences), and carried 16–19 virulence factors. The 27 MSSA isolates were grouped into eight CCs and 12 STs. Seventy-eight percent of the MSSA isolates carried 1–5 different antibiotic resistance genes and carried 5–19 virulence factors. We found *S. aureus* in coastal beach and river waters, anchialine pools, and sand at locations with limited human activity on the island of Hawaiʻi. This may be a public health hazard.

## 1. Introduction

*Staphylococcus aureus* is a facultative pathogen that can be part of the normal flora of humans, as well as some animal species. They have been found in domestic and wild animals, as well as in a variety of environments [1,2,3,4,5,6]. In the United States, *S. aureus* has been isolated from coastal beach waters, freshwaters, and sand in California [7], Florida [5,8,9], Washington State [3,10,11], Ohio [4], the Great Lakes [12], and Hawaiʻi [13,14,15,16]. Methicillin-resistant *Staphylococcus aureus* (MRSA)- and methicillin-susceptible *S. aureus* (MSSA)-related mortality rates have been estimated at 14 and 11 deaths per 100,000 hospitalizations, respectively, in the United States [17]. Previous studies have shown a higher prevalence of MRSA infections in the State of Hawaiʻi compared to the national average [18,19]. Native Hawaiians, Pacific Islanders, and children are disproportionately affected and have a higher infection rate of community-acquired MRSA infections [13,20,21]. A steady increase in the proportion of MRSA, compared to MSSA infections, has been reported in inpatient and outpatient clinics, jails, and nursing homes in Hawaiʻi [13,20,22,23].

Marine waters and beaches have been proposed as possible sources for *S. aureus* infections, but only a few studies have examined the risk to beachgoers in Hawaiʻi and abroad. A correlation between swimmer density and staphylococci isolated from seawater has previously been demonstrated [24]. This resulted in four times higher odds of developing staphylococcal skin infections, as well as increases in respiratory and total illnesses in humans exposed to seawater compared to those who were not; however, the association could not always be confirmed [5,25,26,27].

The aim of the current study was to assess the public health hazard of *S. aureus* found in coastal beach and river waters, anchialine pools, sand, and wastewater on Hawaiʻi Island, Hawaiʻi, by characterizing the isolates using whole-genome sequencing (WGS). This is the first study in the State of Hawaiʻi to characterize MRSA and MSSA isolates using WGS from environmental sources. As this project started, the COVID-19 pandemic began closing tourism to the State of Hawaiʻi which severely reduced beach activities of residents and visitors through lockdowns and limitations on public gatherings. Consequently, this study may underrepresent the full extent of *S. aureus* contamination in coastal and aquatic Hawaiian environments.

## 2. Results

### 2.1. Prevalence of S. aureus

**Figure 1:**
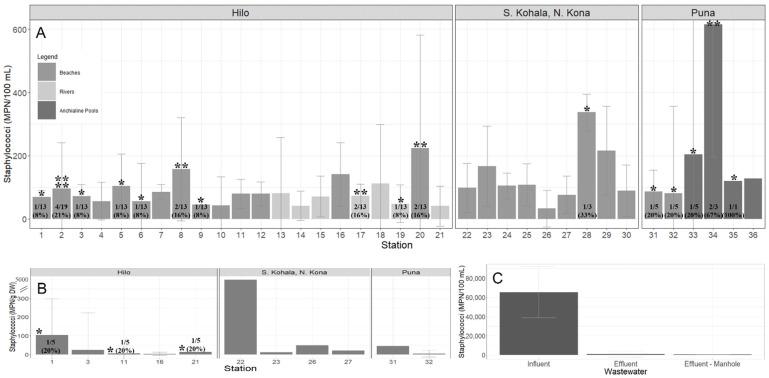
Geometric mean (±SE) of staphylococci collected from the districts of Hilo (stations #1–21), S. Kohala, N. Kona (stations #22–30), and Puna (stations #31–36) from July–December 2020 on Hawaiʻi Island, Hawaiʻi. (**A**) *Staphylococcus* spp. MPN/100 mL counts from water (beach/river/stream/anchialine pool) stations; (**B**) *Staphylococcus* spp. MPN/g DW counts from beach sand stations; (**C**) *Staphylococcus* spp. MPN/100 mL counts from wastewater collected from the Hilo Wastewater Treatment Plant. Detection of *S. aureus* is represented by the *. The number of * is how many different isolates were identified from the same station [ranging from one to four]. The prevalence of *S. aureus*, reported as number of detections in the total sample pool and percent, is shown at the base of each station, if detected.

Ninety-eight percent (354/361) of the samples were positive for *Staphylococcus* spp. Overall, the prevalence of *S. aureus*, MRSA, and MSSA was 7.2% (26/361), 2.2% (8/361) and 5.5% (20/361), respectively, which included 44.4% (16/36) of coastal and freshwater (beach/river/anchialine pool) stations (Table 1). The prevalence of MRSA in coastal beach waters, anchialine pools, and sand was 1.8% (4/225), 10.0% (1/10), and 8.6% (3/35), respectively, and were isolated from the districts of Hilo and Puna. MRSA was not detected in waters at river/stream stations. The prevalence of MSSA in coastal beach and river/stream waters, anchialine pools, and sand was 5.3% (12/225), 3.9% (3/76), 30.0% (3/10), and 5.7% (2/35), respectively. *Staphylococcus* spp. MPN/100 mL counts in water varied 10-fold (Figure 1A). *Staphylococcus* spp. counts in sand were highest at Kehena Beach (station #31) and lowest at Hilo Bay (station #16) (Figure 1B). *S. aureus* isolates were obtained in sand three times, once at Lehia Beach Park (station #1), Reed’s Bay (station #11), and Honoliʻi Beach Park (station #20) (Figure 1B). Wastewater *Staphylococcus* spp. counts varied 10–1000-fold and were higher in influent compared to effluent and manhole samples (Figure 1C). Coastal and freshwater (beach/river/anchialine pool) *S. aureus* isolates were detected at 52.4% (11/21) stations in Hilo, 11.1% (1/9) stations in S. Kohala and N. Kona, and 83.3% (5/6) stations in Puna (Figure 1A). No MRSA or MSSA was detected in wastewater. A total of 36 *S. aureus* isolates were available for genomic analysis.

### 2.2. Genetic Characterization of MRSA and MSSA Isolates

**Figure 2:**
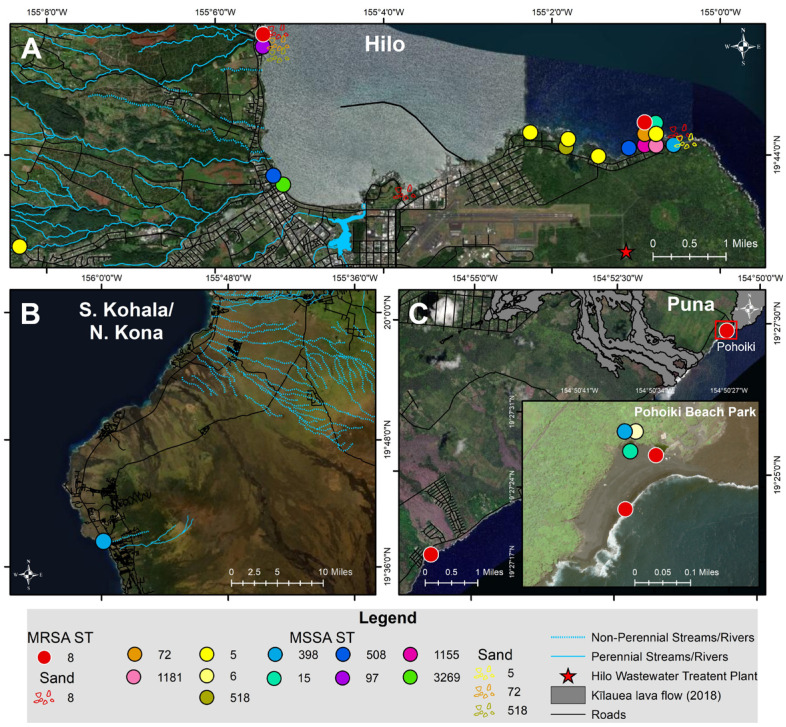
Map of MRSA and MSSA sequence types (ST) isolated from coastal beach and river waters, anchialine pools, and sand from July–December 2020 in the districts of (**A**) Hilo, (**B**) S. Kohala, N. Kona, and (**C**) Puna on Hawaiʻi Island, Hawaiʻi.

**Figure 3:**
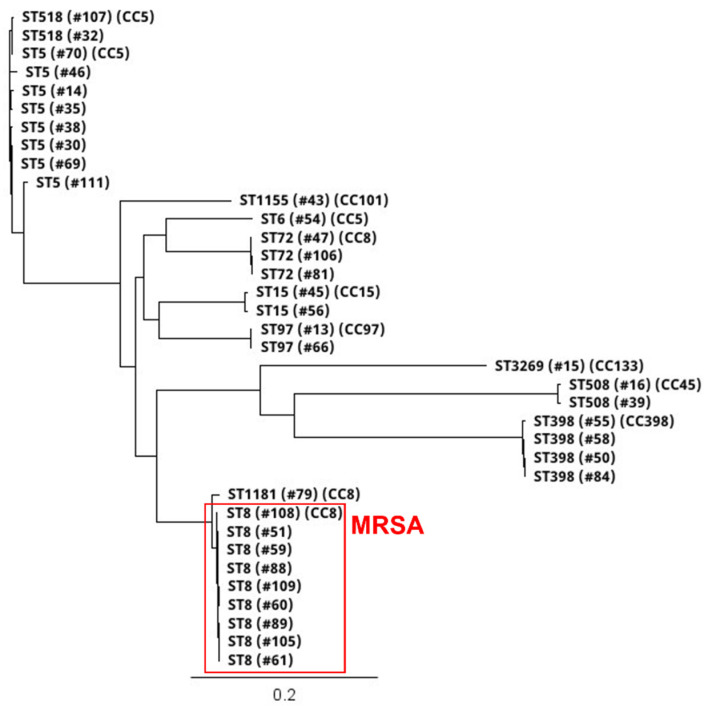
Core genome single nucleotide polymorphism (SNP)-based phylogenetic tree of 12 STs and eight CCs of 36 [nine MRSA, 27 MSSA] isolates collected from coastal beach and river waters, anchialine pools, and sand on Hawaiʻi Island, Hawaiʻi, from July to December 2020. The scale bar denotes genetic distance in number of base substitutions per site. Branch tree labels indicate ST, isolate #, and CC.

**Figure 4:**
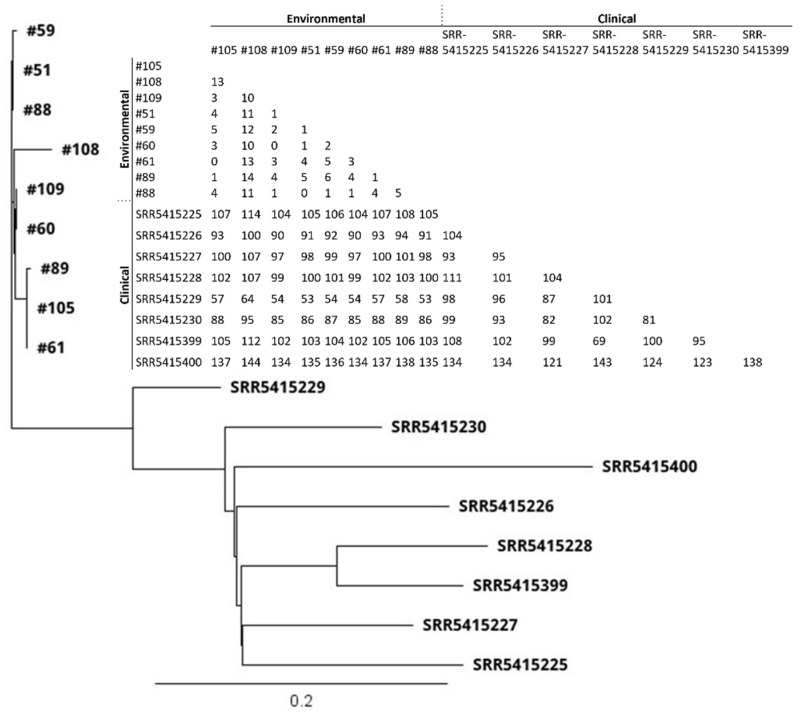
Core genome SNP-based phylogenetic tree and SNP matrix of MRSA (n = 9) isolates obtained from the environment in 2020 from Hawaiʻi Island, Hawaiʻi, and clinical MRSA isolates collected from hospital patients (n = 8) in the State of Hawaiʻi in 2011. Clinical isolate data were obtained from Challagundla et al., 2018 [2] under accession #PRJNA330544. The scale bar denotes genetic distance in number of base substitutions per site.

Thirty-six *S. aureus* isolates included 12 different STs (Figure 2 and Figure 3; Table 2). All nine MRSA isolates [coastal beach water (n = 5), anchialine pool (n = 1), sand (n = 3)] were ST8 (CC8). These isolates were highly clonal based on the core genome SNP analysis (0–16 differences) (Figure 3), despite being isolated from two different districts on the island. The MRSA isolates obtained from the environment in this study were compared with the sequences of clinical MRSA isolates from hospital patients in the State of Hawaiʻi dating back from 2011 [2]. These isolates differed by 54–144 SNPs (Figure 4), and can thus be regarded as clonally related.

Among the 27 MSSA isolates, the most frequent lineages were ST5 (n = 8), ST398 (n = 4), ST72 (n = 3), ST15 (n = 2), ST508 (n = 2), ST97 (n = 2), ST518 (n = 2), and one isolate each of ST6, ST1155, ST3269, and ST1181 (Figure 2 and Figure 3; Table 2). The diversity of STs in each sample was lower in water (beach/river/anchialine pool) (8.7%, 2/23) compared to sand (67%, 2/3) (Table 2), although few *S. aureus* isolates were isolated from sand (n = 3). Within the CC8 group, the three ST72 isolates were genetically related with 5–10 SNP differences (Figure 3), while the ST1181 isolate differed with ~1000 different SNPs. Within the CC5 group, there were three STs: ST5, ST6, and ST518 (Figure 3). Two ST5 isolates were identical, with zero SNP differences, and the other CC5 isolates had 126–631 SNP differences. The two ST518 isolates had six SNP differences between them, whereas the ST6 isolate was genetically distinct from the other CC5 isolates (>10,500 SNP differences). Three of the ST398 isolates were closely related (1–2 SNP differences), whereas one ST398 isolate had 557–558 SNP differences (Figure 3). Two isolates from ST15 (CC15), the one ST508 (CC45), and the ST97 (CC97) isolate had SNP differences of 377, 246, and 7, respectively (Figure 3).

### 2.3. Antibiotic Resistance Genes

The MRSA isolates carried 6–9 different antibiotic resistance genes, including the *mecA* gene (Table 2). All isolates carried aph(3’)-III [aminoglycoside resistance], *erm*(C) [macrolide, lincosamide, streptogramin B resistance], *mph*(C) [macrolide resistance], and *msr*(A) [macrolide resistance] genes. Eight (88.9%) of the isolates carried the *blaZ* [β-lactam resistance] gene. Seven (77.8%) isolates carried *ant(6)-Ia* [aminoglycoside resistance] gene. The other antibiotic resistance genes were identified in fewer isolates (Table 2).

Among the MSSA, 22.2% (6/27) carried no AMR genes, 48.1% (13/27) carried a single AMR gene, 14.8% (4/27) carried two AMR genes, and 14.8% (4/27) carried 4–5 different AMR genes (Table 2). The *blaZ* gene was carried by 70.4% (19/27) of MSSA isolates. Less frequently carried AMR genes are listed in. Among the ST5 CC5 MSSA isolates, 62.5% (5/8) carried the *blaZ* gene and 25% (2/8) carried no AMR genes. All four ST398 MSSA isolates carried the *erm*(T) gene, whereas 75% (3/4) also carried the *blaZ* gene. The ST398 isolates were isolated from all three regions sampled. The ST15 (CC15) (n = 2) and ST508 (CC45) (n = 2) all carried the *blaZ* gene. The ST97 (n = 2), and ST3269 (n = 1) did not carry any AMR genes (Table 2).

### 2.4. Toxin, Exoenzyme, Host Immunity Genes

All MRSA carried the following toxin genes: *hlgA*, *hlgB*, *hlgC*, *lukD*, *lukE*, *lukF-PV*, *lukS-PV*, *sek*, and *seq* (Table 2). One MRSA (isolate #109) also carried the *seg*, *sem*, and *sen* enterotoxin genes and was isolated from sand. All MSSA isolates carried the *hlgA*, *hlgB*, and *hlgC* hemolysin toxin genes. The ST6 (CC8) MSSA (isolate #54) also carried the *sek* and *seq* toxin genes, as identified in the MRSA isolates, but was not found in the other MSSA isolates. The *lukD* and *lukE* toxin genes were identified in 77.8% (21/27) of the MSSA strains. Fifteen (55.6%) of the MSSA isolates contained ≥ 6 enterotoxin genes (Table 2). The most common enterotoxin genes detected were *seg*, *sei*, *sem*, *sen*, *seo*, and *seu*, which were found in 55.6% (15/27) of MSSA isolates. The *tst* gene was detected in both ST508 MSSA isolates and was identified in Hilo at the Wailuku River Mouth (station #17) and at Waiʻōlena (station #5). No enterotoxin genes were found for ST398, ST15, ST97, ST1155, and ST3269 isolates.

All MRSA isolates carried the following exoenzyme genes: *aur*, *splA*, *splB*, and *splE* (Table 2). The *aur* gene was detected in all 27 MSSA isolates. The *splA* gene was carried in 77.8% (21/27) of MSSA isolates. The *splB* gene was carried in 74.1% (20/27) of the MSSA isolates and was missing from ST1181, ST398, and ST508. The *splE* gene was found in 25.9% (7/27) of MSSA isolates and found in ST72, ST6, ST15, and ST1155. MRSA isolates carried the following host immunity genes: *ACME*, *sak*, and *scn* (Table 2). The *scn* gene was found in 96.3% (26/27) of MSSA isolates. The *sak* and *scn* genes were found in 74.1% (20/27) of all sequence types, except for ST398, ST15, and ST3269. ST3269 was the only MSSA isolate that did not carry any host immunity genes.

### 2.5. Plasmids

Among the 36 *S. aureus* strains, nine replicon families (*rep* family) were detected. The *rep* genes were on the same contig as the AMR and/or virulence genes (Table 3). The MRSA isolates contained 2–4 *rep* genes. Among the MSSA isolates, 12 contained no *rep* family genes, 14 contained a single *rep* family, and 1 contained 2 *rep* families (Table 3). The most dominant *rep* families were *rep*_10_ and *rep*_19_ among the MRSA and *rep*_13_ and *rep*_20_ among the MSSA. Among the nine MRSA, all carried the *erm*(C) gene on the *pDLK1* plasmid with *rep*_10,_ seven carried *blaZ* on a *rep*_19_ plasmid, two carried *tet*(K) on a *rep*_17a_ plasmid, and one isolate each carried *msr*(A) on a *rep*_19_ plasmid and *lnu*(A) on a *rep*_21_ plasmid. Among the MSSA, all ST398 and the one ST5 carried the *erm*(T) gene on a plasmid with *rep*_13_. The *tet*(K) gene was associated with *rep*_7a_ for ST1181 (isolate #79) and ST5 (isolate #111). While two MSSA isolates carried the *mph*(C) gene, only one had *rep*_19_. Of the six MSSA isolates that carried the *blaZ* gene, (3/6) were associated with *rep*_16_, (2/6) associated with *rep*_20_, and (1/6) had an unknown *rep* sequence. The *ACME* cluster on a single MRSA isolate was associated with *rep*_7c_. The *aur* virulence gene from three MRSA and two ST72 were associated with *rep*_7c_. Two ST5 carried the *sed* and *sej* toxin genes and were associated with *rep*_20_ and found on the SAP074A plasmid.

## 3. Discussion

In this study, we isolated both MRSA and MSSA from known clonal complexes. The number of positive water samples for *S. aureus* in our study was lower (7.2%) than expected compared to study sites in Florida, which had a 37% positive rate for MSSA and 1% positive rate for MRSA for coastal waters [5]. Similarly other Flordia studies have found more MSSA vs. MRSA in their samples [8,9]. The lower prevalence rate of *S. aureus* compared to the other studies [4,14,15] are most likely a result of the COVID-19 pandemic, whereby the beaches were much less populated with beachgoers and toruists (Hawaiʻi Tourism Authority 2020 https://www.hawaiitourismauthority.org/media/6395/december-2020-visitor-statistics-press-release-final.pdf, accessed on 8 August 2021). This is reflected by studies in Hawaiʻi that had a prevalence up to 92% for *S. aureus* in aquatic environments [14,15,16]. Nevertheless, the strains isolated are of interest as they are most likely contaminated by the local population rather than tourists.

The MRSA, despite being isolated from two different districts on the island (Figure 2), were clonally related and thus, probably indigenous to the region, as they were also clonally related to strains previously isolated in Hawaiʻi State 10 years prior and from a different island [2]. It is clear that this MRSA clone had been circulating on the Hawaiian Islands for a long time. However, limited data are available on MRSA strains circulating in Hawaiʻi. A study conducted in 2003–2004 on the island of Oʻahu, Hawaiʻi typed 12 environmental MRSA isolates via PFGE; seven of the MRSA contained recognizable pulse field types, including: USA100 (4/12), USA300 (1/12), USA1000 (1/12), and USA1100 (1/12) [16]. Another study in 2004 collected 40 MRSA isolates from skin and soft tissue infections from hospital patients; 65.0% (26/40) were classified as USA300 and of those, 84.6% (22/26) were classified as USA300-0114 [13]. Thus, while a diversity of MRSA was found in the mid-2000′s, the dominant MRSA strain in the past decade appears to be the USA300 clone. Nevertheless, given the high infection rate with MRSA in Hawaiʻi [18,19], this indicates that the strain is also widespread amongst the population, as beachgoers during our sampling period were primarily local residents.

The MRSA in this study was the typical USA300 ST8 SCC*mec* IVa community-acquired MRSA, carrying the *PVL* toxin, which is a very prevalent strain in North America and the strains, though an outgroup, cluster together with the USA300 “North American Epidemic” (USA300-NAE) clone [28]. Moreover, an episode of acute increase of MRSA infections in a coastal community in Hawaiʻi has been described in the past [29]. Suggestive of a clear public health hazard associated with this strain circulating on coastal beaches. The USA300 strains have also been detected by Pulse Field Gel Electrophoresis (PFGE) analysis in the past, but only in low numbers [16]. This indicates that this clone has been spreading successfully on the island of Hawaiʻi. Nevertheless, future studies should include human *S. aureus* isolates, in connection with epidemiological data, to examine whether there is a link between recreating in *S. aureus*-contaminated coastal waters and the onset of skin infections. Aside from two older publications, there has been limited indication of an increased risk of developing staphylococcal skin infections as a result of recreating in staphylococci-contaminated waters and beach sand in Hawaiʻi [24,27]. Recreational swimmers in Hawaiʻi may be disproportionately affected by *S. aureus* infections, particularly on Hawaiʻi Island, due to the presence of rocky beaches and coral reefs where skin abrasions/wounds are common. These have previously been identified as an important risk factor for acquiring staphylococcal infections [30]. *S. aureus* has also been found in rivers during storms [15], which attracts surfers who may be disproportionately at risk for acquiring these infections [31].

Overall, this indicates that colonization of *S. aureus* strains may occur at coastal beaches and may, subsequently, spread in the community and cause infections. Additional genomic information of currently circulating strains infecting humans on the island of Hawaiʻi are necessary to bring more clarity to the situation.

The only former WGS data on AMR genes in MRSA from Hawaiʻi indicated that the eight USA300 strains contained 2–9 different antibiotic resistant genes [2]. The *aac*(6’)-*aph*(2’’), and *mupA* genes were not detected in the MRSA isolates in the current study. The AMR genes [*erm*(T), *lnu*(A), *qacD*, *qacG*, and *tet*(K)] found in the current study were not found in the clinical isolates. This suggests that during the period of 10 years, several additional resistance genes were acquired. However, additional studies are necessary to better assess the resistance genes present.

The strains in this study and the formerly isolated eight MRSA [2] carried the same virulence genes, except for the *ACME* cluster, which was carried in only 50% (4/8) of the clinical MRSA compared to 100% (9/9) of the environmental MRSA isolates in the current study. Indeed, the current USA300-NAE strains are characterized by the presence of the *ACME* cluster, while the South American clones are *ACME* negative [28].

The MSSA strains in this study were more diverse, less resistant, and carried fewer virulence genes. The MSSA isolates included closely related ST72 strains from different locations. Similarly, some of the ST5 strains were related, and others were less related to other ST5 strains. The ST518 strains were very related. Moreover, the ST398 strains were nearly the same despite being isolated from all regions sampled. This suggests that these strains may also be endemic in Hawaiʻi, but due to the lack of data, it cannot be stated for certain. The MSSA all belong to well-known clones, such as ST398 MSSA, which is human-associated unlike the MRSA ST398 [32] and carries the *erm*(T) gene, which are not typically found in livestock-associated strains [33]. Furthermore, future in-depth studies should also focus on the epidemiology of MSSA, as these strains account for the majority of invasive *S. aureus* infections in the United States resulting in considerable morbidity and mortality, [34] and may also pose a health risk for beachgoers.

The AMR genes have been shown to be located on different mobile genetic elements in MRSA and MSSA for *blaZ*. Indeed, *blaZ* is located, in general, on a transposon and may jump between different locations [35]. Similarly, the *erm*(T) gene, originally described in an MSSA ST398 [36], has been found on different plasmids, though to our knowledge never on USA300 strains. Unfortunately, it is unknown what the prevalence of ST398 is in Hawaiʻi. However, we found it rather abundantly in this study, and it is a human-associated strain. It appears to be quite prevalent, and the *erm*(T) gene might have been transferred from ST398 to ST8, ST5, and ST518 (Table 2).

Few plasmids are common between the MRSA and MSSA, though it is also clear that the MRSA population is quite clonal and may represent a bias in the diversity. Nevertheless, it seems that the USA300 clone is also the predominant clone on the island. However, there are also clear indications of the exchanges of *rep*_7c_, *rep*_7a_ and *rep*_19_ plasmids. While these plasmids have the same replicon type, they do not always carry the same resistance genes. It is clear that the MRSA are able to more easily acquire plasmids as they are overwhelmingly present in the MRSA opposed to MSSA strains.

## 4. Materials and Methods

### 4.1. Study Locations and Sampling

**Figure 5:**
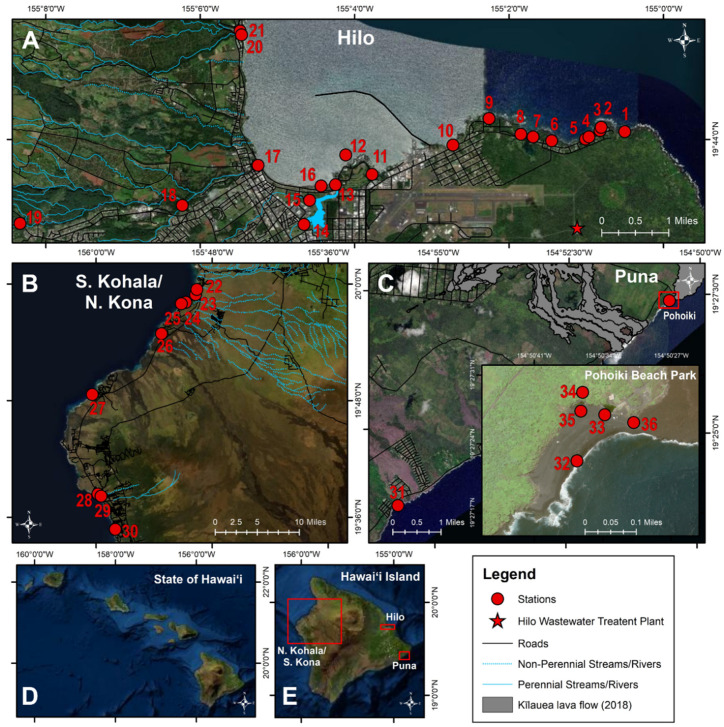
Map of water sampling stations. Sampling was conducted at 36 water (beach/river/stream/anchialine pool) and sand stations in the districts of (**A**) Hilo (station #1–21), (**B**) S. Kohala, N. Kona (station #22–30), and (**C**) Puna (station #31–36) on (**D**) Hawaiʻi Island, (**E**) Hawaiʻi from June to December 2020.

Water and sand samples were collected multiple times at 36 stations across the three most populous districts in Hawaiʻi County, including the most populated district, Hilo, and the less populated ones of South Kohala, North Kona, and Puna (Figure 5). Hilo stations were chosen adjacent to and within the Hilo Bay watershed, spanning ~12 km of coastline, including the Wailuku and Wailoa Rivers and Honoliʻi Stream. A majority of households in Hilo rely on decentralized wastewater management, where ~8700 cesspools leak ~21.2 million liters/day into groundwater, which contaminates nearshore coastal waters [37]. In Hilo, there is a wastewater treatment plant that utilizes a series of gravity-fed systems with pump stations and provides primary and secondary treatment, where effluent is discharged offshore via an ocean outfall. As a result of heavy rainfall, cesspool seepage and sewage overflows periodically occur representing a potential health hazard and thus, sewage was sampled. Twenty-one stations were selected, which included 14 State and county beaches (stations #1–12, #16, #20; 188 samples) seven river/stream stations (76 samples) along the Wailoa River (stations #13–15), Wailuku River (stations #17–19), and Honoliʻi Stream (station #21). Sand was collected at five stations in Hilo (stations #1–2, #11, #16, #20) each on five occasions (25 samples). Wastewater influent, effluent, and treated effluent collected from a manhole was collected monthly over five months (15 samples) from the Hilo Wastewater Treatment Plant (Figure 5A).

The districts of S. Kohala and N. Kona and are located on the western side of Hawaiʻi Island, dominated by resort developments. Nine county and private beach stations were sampled (stations #22–30; 27 samples). Sand was collected once at four stations in S. Kohala and N. Kona (stations #22–23, #26–27) (Figure 5B).

Puna is a rapidly developing district located southeast of Hilo, and in 2018, a black sand beach and numerous anchialine pools formed at a popular surfing spot (Pohoiki) following a volcanic eruption [38]. Two county beaches (stations #31–32; 10 samples) and four anchialine pools (stations #33–36; 10 samples) were sampled. Sand was collected at two stations in Puna, each on three occasions (stations #31–32) (6 samples) (Figure 5C).

A total of 361 samples were collected and processed from coastal beach waters (n = 225), rivers/stream (n = 76), anchialine pools (n = 10), sand (n = 35), and wastewater (n = 15) between July–December 2020 (Figure 5). Liquid samples were collected in sterile, acid-washed polypropylene plastic bottles between 6 AM and noon, stored on ice, and processed within 6 h of collection. Sand samples were collected in sterile, polypropylene bottles using a metal spade 1–2” deep, within 1 m of the high tide line, transported on ice, and processed within 6 h.

### 4.2. Isolation and Quantification of Staphylococci

Water samples were cultured for *Staphylococcus* spp. using broth enrichment (1.5 X m *Staphylococcus* broth (Becton, Dickinson, and Company, Sparks, MD, USA), 75 µg/mL polymyxin B, and 0.01% potassium tellurite) in Quanti-Tray 2000^®^ (IDEXX Laboratories, Westbrook, ME, USA) and incubated for 72 h at 37 °C, as previously described [3]. Positive wells contained a black precipitate [3,10]. The number of positive wells were counted and the presumptive *Staphylococcus* spp. MPN/100 mL was calculated using the MPN IDEXX chart and adjusted for dilution. From each Quanti-Tray 2000^®^ sample, ≤1 mL of broth from positive wells were placed into a 1.5 mL microcentrifuge tube and vortexed for 15 s. An aliquot was inoculated onto both mannitol salt agar (Becton, Dickinson, and Company) and Oxacillin Resistance Selective Agar Base (ORSAB) with supplement (Thermo Fisher Scientific^TM^ Oxoid^TM^ Products, Lenexa, KS, USA), and incubated at 37 °C to screen for MRSA and MSSA, respectively. Presumptive MRSA/MSSA colonies were inoculated onto blood agar (Tryptone Soya agar with sheep blood) (Thermo Fisher Scientific^TM^ Remel^TM^ Products), incubated at 37 °C for 18–24 h in a candle extinction jar and screened for β-hemolysis. Presumptive MRSA and MSSA isolates were identified using the Staphaurex^TM^ latex agglutination test kit (Thermo Fisher Scientific^TM^ Remel^TM^ Products). Presumptive MRSA isolates were biochemically identified using the penicillin-binding protein (PBP2) latex agglutination test kit (Thermo Fisher Scientific^TM^ Remel^TM^ Products). A single MRSA/MSSA isolate was stored at 4 °C until further characterized. In seven (30%) of the 23 positive water (beach/river/anchialine pool) samples, two different wells contained *S. aureus,* which were characterized to assess diversity in the STs (Table 2).

Sand samples were processed using modified methods, as previously described [39]. Briefly, 10 g of wet sand from each station were shaken with 200 mL of 0.15 M NaCl for two min at 100 rpm where 25 mL of the supernatant was transferred into a sterile bottle, and sterile water added for a final volume of 50 mL. This volume was then combined with 50 mL of the enrichment broth and processed, as described above in IDEXX trays [3]. Up to 20 mL of wastewater was transferred; sterile water was added for a final volume of 50 mL. Samples were then combined with 50 mL of enrichment broth and placed in Quanti-Tray 2000^®^ and processed, as previously described [3]. In two (67%) of the three positive sand samples, 2–3 different wells contained *S. aureus,* which were characterized to assess diversity in the STs (Table 2).

### 4.3. WGS Assembly and Analysis

From 26 *S. aureus* samples, nine MRSA and 27 MSSA isolates were characterized using WGS. Library preparation was performed using the Illumina DNA Prep Kit (Illumina, Inc., San Diego, CA, USA) and sequenced on a MiSeq machine (Illumina, Inc.) using a paired end 2 × 250 bp sequencing strategy, following standard Illumina protocols (Illumina, Inc., San Diego, CA, USA). Raw reads were quality filtered and trimmed using Trimmomatic (v0.39.0) [40] with LEADING/TAILING 3 and MINLENGTH 36 used as default parameters. FASTQ files were imported into Geneious Prime v2021.1 and de novo assembled using the SPAdes genome assembler (v3.13.0) [41] in careful mode. Sequence types were determined using the *S. aureus* MLST 2.0 database (https://cge.cbs.dtu.dk/services/MLST/, accessed on 1 February 2021), SCC*mec* type elements determined using SCC*mec*Finder 1.2 (https://cge.cbs.dtu.dk/services/SCCmecFinder/, accessed on 1 February 2021). ResFinder 4.1 (https://cge.cbs.dtu.dk/services/ResFinder/, accessed on 1 February 2021) and VirulenceFinder 2.0 (https://cge.cbs.dtu.dk/services/VirulenceFinder/, accessed on 1 February 2021) using identity thresholds of 90% and a minimum length of 60% for the detection of resistance and virulence genes. The presence of plasmids was assessed using MobileElementFinder, using default settings (https://cge.cbs.dtu.dk/services/MobileElementFinder/, accessed on 1 February 2021). Core genome pairwise single nucleotide polymorphism (SNP) was assessed using CSI Phylogeny 1.4 (https://cge.cbs.dtu.dk/services/CSIPhylogeny/, accessed on 1 February 2021) using default settings. The following isolates were used as reference strains for phylogenetic analyses: MRSA ST8 (CC8) (accession #SAMN18836599), MSSA ST 5 (CC5) (accession #SAMN18824826), and MSSA ST398 (CC398) (accession #SAMN18824846). Phylogenetic trees were visualized using Geneious Prime v2021.1 (Figure 2 and Figure 3). All WGS data were deposited in NCBI GenBank under project PRJNA721726 with the following accession numbers: SAMN18824809–SAMN18824812, SAMN18824826, SAMN18824828, SAMN18824831, SAMN18824834–SAMN18824835, SAMN18824839, SAMN18824841, SAMN18824843, SAMN18824846–SAMN18824847, SAMN18824850–SAMN18824852, SAMN18824854–SAMN18824857, SAMN18836577, SAMN18836580–SAMN18836581, SAMN18836590–SAMN18836592, SAMN18836595, SAMN18836599–SAMN18836600, SAMN18836616–SAMN18836620, and SAMN18836622.

### 4.4. Prevalence of S. aureus

Descriptive statistics (geometric mean, standard error, minimum, maximum) was carried out in R Studio (v.4.0.5).

## 5. Conclusions

The beaches in Hawaiʻi Island contaminated both MRSA and MSSA. The MRSA in this study were the typical USA300 strains, which are likely circulating in the community. The MRSA strains were very clonal. The MSSA strains isolated in this study were less resistant and have less virulence traits; however, there is also a lot of clonality amongst the strains. This indicates that the beaches are a potential health risk for both MRSA and MSSA infections in humans.

## Figures and Tables

**Table 1:** Distribution and prevalence of *Staphylococcus aureus* samples (n = 26) and isolates (n = 36) characterized from environmental sources. Methicillin-resistant *S. aureus* (MRSA); methicillin-susceptible *S. aureus* (MSSA).

District	Source	# Stations	# Quanti-Tray 2000^®^ Samples	# MRSA Isolates	# MSSA Isolates	# *S. aureus* Isolates	# Quanti-Tray 2000^®^ Samples Prevalence (%)		
							MRSA	MSSA	*S. aureus*
**Hilo**	Beaches	14	188	2	17	19	2 (1.1)	11 (5.9)	13 (6.9)
	Rivers/Stream	7	76	0	3	3	0 (0.0)	3 (3.9)	3 (3.9)
	Sand	5 *	25	3	3	6	3 (12.0)	2 (8.0)	3 (12.0)
	Wastewater	N/A	15	0	0	0	0 (0.0)	0 (0.0)	0 (0.0)
**S. Kohala,** **N. Kona**	Beaches	9	27	0	1	1	0 (0.0)	1 (3.7)	1 (3.7)
	Sand	4 *	4	0	0	0	0 (0.0)	0 (0.0)	0 (0.0)
**Puna**	Beaches	2	10	3	0	3	2 (20.0)	0 (0.0)	2 (20.0)
	Anchialine Pools	4	10	1	3	4	1 (10.0)	3 (30.0)	4 (40.0)
	Sand	2 *	6	0	0	0	0 (0.0)	0 (0.0)	0 (0.0)
**Summary**									
**Hilo,** **S. Kohala, N. Kona, Puna**	Beaches	25	225	5	18	23 ^d^	4 (1.8)	12 (5.3)	16 (7.1)
	Rivers/Stream	7	76	0	3	3	0 (0.0)	3 (3.9)	3 (3.9)
	Anchialine Pools	4	10	1	3	4	1 (10.0)	3 (30.0)	4 (40.0)
	Sand	11 *	35	3	3	6 ^e^	3 (8.6)	2 (5.7)	3 (8.6)
	Wastewater	N/A	15	0	0	0	0 (0.0)	0 (0.0)	0 (0.0)
	Total	36	361	9 ^a^	27 ^b^	36 ^c^	8 (2.2)	20 (5.5)	26 (7.2)

* Sand samples were taken from the same beach station and therefore, not included in the total. ^a^ Nine MRSA isolates were obtained from six samples, plus two that have both MRSA and MSSA isolated. ^b^ Twenty-seven MSSA isolates were obtained from 18 samples, plus two that had MRSA and MSSA isolated. ^c^ Thirty-six *S. aureus* isolates were obtained from 26 samples. ^d^ Fourteen *S. aureus* isolates were obtained from seven samples. From each Quanti-tray 2000^®^ sample, two MSSA isolates were characterized: Lehia Beach Park (station #1); Richardson’s Beach Park (station #2; 08/21/2020); Richardson’s Beach Park (station #2; 11/14/2020), Kealoha Beach Park (station #8); Onekahakaha Beach Park (station #9); Honoliʻi Beach Park (station #20). Two MRSA isolates were obtained from Pohoiki Beach (station #32). See Table 2 for additional details. ^e^ Six *S. aureus* isolates were obtained from three samples. This included: one MRSA and one MSSA from Lehia Beach Park (station #1); one MRSA and two MSSA from Honoliʻi Beach Park (station #20); one MSSA from Reed’s Bay (station #11). See Table 2 for additional details.

**Table 2:** Genomic characterization of MRSA (n = 9) and MSSA (n = 27) isolates collected from coastal beach and river waters, anchialine pools, and beach sand from July–December 2020 in the districts of Hilo, N. Kona, and Puna on Hawaiʻi Island, Hawaiʻi.

Station	District	Source	Date	Isolate #	MRSA/MSSA	ST ^b^	CC ^c^	Antimicrobial Resistance ^d^	Virulence Gene Profile ^e^			Plasmids
									Toxin	Exoenzyme	Host Immunity	
**Lehia Beach Park** **(** **station** **#1)**	Hilo	sand	12/12/2020	108	MRSA ^a^	8 ^f^	8	*aph*(3’)-*III*, *blaZ*, *erm*(C), *erm*(T)*, mecA*, *mph*(C), *msr*(A)	*hlgA*, *hlgB*, *hlgC*, *lukD*, *lukE*, *lukF*-*PV*, *lukS*-*PV*, *sek*, *seq*	*aur*, *splA*, *splB*, *splE*	*ACME*, *sak*, *scn*	*rep*_10_, *rep*_19_
**Richardson’s Beach Park** **(station #3)**	Hilo	beach water	09/04/2020	60	MRSA ^a^	8	8	*aph*(3’)-*III*, *ant*(6)-*Ia, blaZ*, *erm*(C), *mecA*, *mph*(C), *msr*(A)	*hlgA*, *hlgB*, *hlgC*, *lukD*, *lukE*, *lukF*-*PV*, *lukS*-*PV*, *sek*, *seq*	*aur*, *splA*, *splB*, *splE*	*ACME*, *sak*, *scn*	*rep*_10_, *rep*_19_
**Reed’s Bay** **(station** **#11)**	Hilo	sand	12/14/2020	105	MRSA ^a^	8	8	*aph*(3’)-*III*, *blaZ*, *erm*(C), *mecA*, *mph*(C), *msr*(A), *tet*(K)	*hlgA*, *hlgB*, *hlgC*, *lukD*, *lukE*, *lukF*-*PV*, *lukS*-*PV*, *sek*, *seq*	*aur*, *splA*, *splB*, *splE*	*ACME*, *sak*, *scn*	*rep*_7a_, *rep*_7c_, *rep*_10_, *rep*_19_
**Honoliʻi Beach Park** **(** **station #20)**	Hilo	beach water	08/15/2020	59	MRSA ^a^	8	8	*aph*(3’)-*III*, *ant*(6)-*Ia, blaZ*, *erm*(C), *mecA*, *mph*(C), *msr*(A)	*hlgA*, *hlgB*, *hlgC*, *lukD*, *lukE*, *lukF*-*PV*, *lukS*-*PV*, *sek*, *seq*	*aur*, *splA*, *splB*, *splE*	*ACME*, *sak*, *scn*	*rep*_7c_, *rep*_10_, *rep*_19_
**Honoliʻi Beach Park** **(** **station #20)**	Hilo	sand	12/14/2020	109	MRSA ^a^	8 ^g^	8	*aph*(3’)-*III*, *ant*(6)-*Ia, blaZ*, *erm*(C), *mecA*, *mph*(C), *msr*(A), *qacG*, *tet*(K)	*hlgA*, *hlgB*, *hlgC*, *lukD*, *lukE*, *lukF*-*PV*, *lukS*-*PV*, *seg*, *sem*, *sen*, *sek*, *seq*	*aur*, *splA*, *splB*, *splE*	*ACME*, *sak*, *scn*	*rep*_7a_, *rep*_10_, *rep*_19_
**Kehena** **(station** **#31)**	Hilo	beach water	08/20/2020	61	MRSA ^a^	8	8	*aph*(3’)-*III*, *ant*(6)-*Ia, erm*(C), *mecA*, *mph*(C), *msr*(A)	*hlgA*, *hlgB*, *hlgC*, *lukD*, *lukE*, *lukF*-*PV*, *lukS*-*PV*, *sek*, *seq*	*aur*, *splA*, *splB*, *splE*	*ACME*, *sak*, *scn*	*rep*_7c_, *rep*_10_, *rep*_19_
**Pohoiki Beach** **(station #32)**	Puna	beach water	08/20/2020	51	MRSA ^a^	8 ^h^	8	*aph*(3’)-*III, ant*(6)-*Ia, blaZ, erm*(C), *mecA, mph*(C), *msr*(A)	*hlgA*, *hlgB*, *hlgC*, *lukD*, *lukE*, *lukF*-*PV*, *lukS*-*PV*, *sek*, *seq*	*aur*, *splA*, *splB*, *splE*	*ACME*, *sak*, *scn*	*rep*_10_, *rep*_19_
**Pohoiki Beach** **(station #32)**	Puna	beach water	08/20/2020	88	MRSA ^a^	8 ^h^	8	*aph*(3’)-*III, ant*(6)-*Ia, blaZ, erm*(C), *mecA, mph*(C), *msr*(A), *qacD*	*hlgA*, *hlgB*, *hlgC*, *lukD*, *lukE*, *lukF*-*PV*, *lukS*-*PV*, *sek*, *seq*	*aur*, *splA*, *splB*, *splE*	*ACME*, *sak*, *scn*	*rep*_10_, *rep*_19_
**Pohoiki Harbor** ** (station #33)**	Puna	anchialine pool	12/16/2020	89	MRSA ^a^	8	8	*aph*(3’)-*III, ant*(6)-*Ia, blaZ, erm*(C), *lnu*(A), *mecA, mph*(C), *msr*(A), *qacD*	*hlgA*, *hlgB*, *hlgC*, *lukD*, *lukE*, *lukF*-*PV*, *lukS*-*PV*, *sek*, *seq*	*aur*, *splA*, *splB*, *splE*	*ACME*, *sak*, *scn*	*rep*_10_, *rep*_19_, *rep*_21_
**Richardson’s Beach Park** **(station #2)**	Hilo	beach water	11/14/2020	47	MSSA	72 ^i^	8	*blaZ*	*hlgA*, *hlgB*, *hlgC*, *lukD*, *lukE*, *sec*, *seg*, *sei*, *sel*, *sem*, *sen*, *seo*, *seu*	*aur*, *splA*, *splB*, *splE*	*sak*, *scn*	-
**Richardson** **’** **s Beach Park** **(station #2** **)**	Hilo	beach water	11/14/2020	81	MSSA	72 ^i^	8	*blaZ*, *lnu*(A)	*hlgA*, *hlgB*, *hlgC*, *lukD*, *lukE*, *sec*, *seg*, *sei*, *sel*, *sem*, *sen*, *seo*, *seu*	*aur*, *splA*, *splB*, *splE*	*sak*, *scn*	*rep*_7c_
**Honoliʻi Beach Park ** **(station #2)**	Hilo	sand	12/14/2020	106	MSSA	72 ^g^	8	*blaZ*	*hlgA*, *hlgB*, *hlgC*, *lukD*, *lukE*, *sec*, *seg*, *sei*, *sel*, *sem*, *sen*, *seo*, *seu*	*aur*, *splA*, *splB*, *splE*	*sak*, *scn*	*rep*_7c_
**Richardson’s Beach Park** **(station #2)**	Hilo	beach water	08/21/2020	79	MSSA	1181 ^j^	8	*aph*(3’)-*III*, *ant*(6)-*Ia*, *blaZ*, *tet*(K)	*hlgA*, *hlgB*, *hlgC*, *lukD*, *lukE*, *sep*	*aur*, *splA*	*sak*, *scn*	*rep*_7a_
**Lehia Beach Park** ** (station #1)**	Hilo	sand	12/14/2020	111	MSSA	5 ^f^	5	*blaZ*, *erm*(T), *mph*(C), *tet*(K)	*hlgA*, *hlgB*, *hlgC*, *lukD*, *lukE*, *seg*, *sei*, *sem*, *sen*, *seo*, *seu*	*aur*, *splA*, *splB*	*sak*, *scn*	*rep*_7a_, *rep*_13_
**Lalakea** ** (station #6)**	Hilo	beach water	08/21/2020	38	MSSA	5	5	-	*hlgA*, *hlgB*, *hlgC*, *lukD*, *lukE*, *seg*, *sei*, *sem*, *sen*, *seo*, *seu*	*aur*, *splA*	*sak*, *scn*	-
**Kealoha Beach Park** **(** **station #8)**	Hilo	beach water	10/31/2020	35	MSSA	5	5	*blaZ*	*hlgA*, *hlgB*, *hlgC*, *lukD*, *lukE*, *sed*, *seg*, *sei*, *sej*, *sem*, *sen*, *seo*, *ser*, *seu*	*aur*, *splA*, *splB*	*sak*, *scn*	*rep*_20_
**Kealoha Beach Park** **(** **station #8)**	Hilo	beach water	07/03/2020	70	MSSA	5 ^k^	5	*lnu*(A)	*hlgA*, *hlgB*, *hlgC*, *lukD*, *lukE, seg*, *sei*, *sem*, *sen*, *seo*, *seu*	*aur*, *splA*, *splB*	*sak*, *scn*	-
**Onekahakaha Beach Park** **(** **station #9)**	Hilo	beach water	08/21/2020	30	MSSA	5 ^l^	5	*blaZ*	*hlgA*, *hlgB*, *hlgC*, *lukD*, *lukE*, *seg*, *sei*, *sem*, *sen*, *seo*, *seu*	*aur*, *splA*, *splB*	*sak*, *scn*	*rep*_16_
**Onekahakaha Beach Park** **(** **station #9)**	Hilo	beach water	08/21/2020	69	MSSA	5 ^l^	5	-	*hlgA*, *hlgB*, *hlgC*, *lukD*, *lukE*, *seg*, *sei*, *sem*, *sen*, *seo*, *seu*	*aur*, *splA*, *splB*	*sak*, *scn*	-
**Wailuku River–Upper** **(** **station #19)**	Hilo	river water	10/31/2020	14	MSSA	5	5	*blaZ*	*hlgA*, *hlgB*, *hlgC*, *lukD*, *lukE*, *sed*, *seg*, *sei*, *sej*, *sem*, *sen*, *seo*, *ser*, *seu*	*aur*, *splA*, *splB*	*sak*, *scn*	*rep*_20_
**Richardson’s Beach Park** **(** **station #3)**	Hilo	beach water	10/31/2020	46	MSSA	5	5	*aph*(3’)*-III*, *ant*(6)*-Ia*, *blaZ*, *mph*(C), *msr*(A)	*hlgA*, *hlgB*, *hlgC*, *lukD*, *lukE*, *seg*, *sei*, *sem*, *sen*, *seo*, *seu*	*aur*, *splA*, *splB*	*sak*, *scn*	*rep*_19_
**Pohoiki** ** (** **station #34)**	Puna	anchialine pool	09/18/2020	54	MSSA	6	5	*blaZ*	*hlgA*, *hlgB*, *hlgC*, *lukD*, *lukE*, *sea*, *seb*, *sek*, *seq*	*aur*, *splA*, *splB*, *splE*	*sak*, *scn*	*rep*_16_
**Kealoha Beach Park** **(station** **#8)**	Hilo	beach water	07/03/2020	32	MSSA	518 ^k^	5	-	*hlgA*, *hlgB*, *hlgC*, *lukD*, *lukE*, *seg*, *sei*, *sem*, *sen*, *seo*, *seu*	*aur*, *splA*, *splB*	*sak*, *scn*	-
**Honoliʻi Beach Park** ** (station #20)**	Hilo	sand	12/14/2020	107	MSSA	518 ^g^	5	*blaZ*, *erm*(T), *mph*(C), *tet*(K)	*hlgA*, *hlgB*, *hlgC*, *lukD*, *lukE*, *seg*, *sei*, *sem*, *sen*, *seo*, *seu*	*aur*, *splA*, *splB*	*sak*, *scn*	-
**Lehia Beach Park** **(** **station #1)**	Hilo	beach water	10/31/2020	50	MSSA	398 ^m^	398	*blaZ*, *erm*(T)	*hlgA*, *hlgB*, *hlgC*	*aur*	*scn*	*rep*_13_
**Lehia Beach Park** **(station #1)**	Hilo	beach water	10/31/2020	84	MSSA	398 ^m^	398	*blaZ*, *erm*(T)	*hlgA*, *hlgB*, *hlgC*	*aur*	*scn*	*rep*_13_
**Kailua Pier** **(** **station #28)**	N. Kona	beach water	10/17/2020	58	MSSA	398	398	*blaZ*, *erm*(T)	*hlgA*, *hlgB*, *hlgC*	*aur*	*scn*	*rep*_13_
**Pohoiki** **Beach** **(** **station #35)**	Puna	anchialine pool	12/16/2020	55	MSSA	398	398	*erm*(T)	*hlgA*, *hlgB*, *hlgC*	*aur*	*scn*	*rep*_13_
**Richardson’s Beach Park** **(** **station #3)**	Hilo	beach water	08/21/2020	45	MSSA	15 ^j^	15	*blaZ*	*hlgA*, *hlgB*, *hlgC*, *lukD*, *lukE*	*aur*, *splA*, *splB*, *splE*	*scn*	*rep*_16_
**Pohoiki** **Beach** **(** **station #34)**	Puna	anchialine pool	12/16/2020	56	MSSA	15	15	*blaZ*	*hlgA*, *hlgB*, *hlgC*, *lukD*, *lukE*	*aur*, *splA*, *splB*, *splE*	*scn*	-
**Waiʻōlena Beach Park** **(** **station #5)**	Hilo	beach water	07/31/2020	39	MSSA	508	45	*blaZ*	*hlgA*, *hlgB*, *hlgC*, *sec*, *seg*, *sei*, *sel*, *sem*, *sen*, *seo*, *seu*, *tst*	*aur*	*sak*, *scn*	-
**Wailuku River Estuary** ** (station #17)**	Hilo	river water	12/14/2020	16	MSSA	508	45	*blaZ*	*hlgA*, *hlgB*, *hlgC*, *sec*, *seg*, *sei*, *sel*, *sem*, *sen*, *seo*, *seu*, *tst*	*aur*	*sak*, *scn*	*rep*_US5_
**Honoliʻi Beach Park** **(** **station #20)**	Hilo	beach water	09/04/2020	13	MSSA	97 ^n^	97	-	*hlgA*, *hlgB*, *hlgC*, *lukD*, *lukE*	*aur*, *splA*, *splB*	*sak*, *scn*	-
**Honoliʻi Beach Park** **(** **station #20)**	Hilo	beach water	09/04/2020	66	MSSA	97 ^n^	97	-	*hlgA*, *hlgB*, *hlgC*, *lukD*, *lukE*	*aur*, *splA*, *splB*	*sak*, *scn*	-
**Richardson’s Beach Park** **(** **station #2)**	Hilo	beach water	10/31/2020	43	MSSA	1155	101	*blaZ*	*hlgA*, *hlgB*, *lukD, lukE*	*aur*, *splA*, *splB*, *splE*	*sak*, *scn*	-
**Wailuku River Estuary** **(** **station #17)**	Hilo	river water	08/15/2020	15	MSSA	3269	133	-	*hlgA*, *hlgB*, *hlgC*, *lukD*, *lukE*	*aur*, *splA*, *splB*	-	-

^a^ MRSA: SCC*mec* type: IVa. ^b^ MLST type determined by the allelic profile of seven *S. aureus* housekeeping genes: *arc*, *aro*, *glp*, *gmk*, *pta*, *tpi*, and *ygi*. ^c^ Clonal Complex (CC). ^d^ Antibiotic resistant genes (corresponding drug class): *blaZ* & *mecA* (beta-lactam), *aph*(3’)-*III* & *ant*(6)-*Ia* (aminoglycoside), *erm*(C) & *erm*(T) (macrolide, lincosamide, streptogrammin B), *lnu*(A) (lincosamide), *msr*(A) (macrolide), *tet*(K) (tetracycline), and *qacD* & *qacG* (quaternary ammonium compounds). ^e^ Virulence factor genes encoding toxins: *hlgA* (gamma-hemolysin chain II precursor), *hlgB* (gamma-hemolysin component B precuror), *hlgC* (gamma-hemolysisn component C), *lukD* (leukocidin D component), *lukE* (leukocidin D component), *lukF*-*PV* (Pantom Valentine leukocidin F component), *lukS*-*PV* (Pantom Valentine leukocidin S component), and enterotoxins (*sea*, *seb*, *sec*, *sed*, *seg*, *sei*, *sej*, *sek*, *sel*, *sem*, *sen*, *seo*, *seq*, *ser*, *seu*), *tst* (toxic shock syndrome toxin-1); exoenzymes: *aur* (aureolysin), serine proteases (*splA*, *splB*, *splE*); host immunity: *ACME* (arginine catabolic mobile element), *sak* (staphylokinase), *scn* (staphylococcal complement inhibition). ^f^ Isolates were chosen from the same Quanti-Tray 2000^®^ sample containing two different STs (ST8, ST5); source: sand. ^g^ Isolates were chosen from the same Quanti-Tray 2000^®^ sample containing three different STs (ST8, ST72, ST518); source: sand. ^h^ Isolates were chosen from the same Quanti-Tray 2000^®^ sample containing the same ST (ST8); source: beach water. ^i^ Isolates were chosen from the same Quanti-Tray 2000^®^ sample containing the same ST (ST72); source: beach water. ^j^ Isolates were chosen from the same Quanti-Tray 2000^®^ sample containing two different STs (ST1181, ST5); source: beach water. ^k^ Isolates were chosen from the same Quanti-Tray 2000^®^ sample containing two different STs (ST5, ST518); source: beach water. ^l^ Isolates were chosen from the same Quanti-Tray 2000^®^ sample containing the same ST (ST5); source: beach water. ^m^ Isolates were chosen from the same Quanti-Tray 2000^®^ sample containing the same ST (ST398); source: beach water. ^n^ Isolates were chosen from the same Quanti-Tray 2000^®^ sample containing the same ST (ST97); source: beach water.

**Table 3:** Association of antibiotic resistance genes and/or virulence genes, and replicon families (*rep* families) with plasmids.

Station	District	Source	Date	Isolate #	MRSA/MSSA	ST ^a^	CC ^b^	Antibiotic Resistance Genes (*rep* Family)	Virulence Genes (*rep* Family)	Plasmid	
										Name	Accession #
**Lehia Beach Park** **(station #1)**	Hilo	sand	12/12/2020	108	MRSA	8	8	*blaZ (rep*_19_) *erm*(C) *(rep*_10_)	-	pWBG759 pDLK1	GQ900401.1 GU562624.1
**Richardson’s Beach Park** **(station #3)**	Hilo	beach water	09/04/2020	60	MRSA	8	8	*blaZ (rep*_19_) *erm*(C) (*rep*_10_)	*ACME* (*rep*_7c_)	TCH60 pDLK1 -	CP002111.1 GU562624.1 KF175393.1
**Reed’s Bay** **(station #11)**	Hilo	sand	12/14/2020	105	MRSA	8	8	*blaZ* (*rep*_19_) *erm*(C) (*rep*_10_) *mecA* (*rep*_7c_) *tet*(K) (*rep*_7a_)	*aur* (*rep*_7c_)	pWBG759 pDLK1 - -	GQ900401.1 GU562624.1 BX571857.1 AB037671.1
**Honoliʻi Beach Park** **(station #20)**	Hilo	beach water	08/15/2020	59	MRSA	8	8	*blaZ* (*rep*_19_) *erm*(C) (*rep*_10_) *mecA* (*rep*_7c_)	*aur* (*rep*_7c_)	TCH60 pDLK1 -	CP002111.1 GU562624.1 BX571857.1
**Honoliʻi Beach Park** **(station #20)**	Hilo	sand	12/14/2020	109	MRSA	8	8	*blaZ* (*rep*_19_) *erm*(C) (*rep*_10_) *tet*(K) (*rep*_7a_)	-	TCH60 pDLK1 -	CP002111.1 GU562624.1 AB037671.1
**Kehena** **(station #31)**	Hilo	beach water	08/20/2020	61	MRSA	8	8	*erm*(C) (*rep*_10_) *mecA* (*rep*_7c_) *mph*(C) (*rep*_19_)	*aur* (*rep*_7c_)	pDLK1 - pWBG759	GU562624.1 BX571857.1 GQ900401.1
**Pohoiki Beach** **(** **station #32)**	Puna	beach water	08/20/2020	51	MRSA	8	8	*msr*(A) (*rep*_19_) *erm*(C) (*rep*_10_)	-	TCH60 pDLK1	CP002111.1 GU562624.1
**Pohoiki Beach** **(** **station #32)**	Puna	beach water	08/20/2020	88	MRSA	8	8	*blaZ* (*rep*_19_) *erm*(C) (*rep*_10_)	-	pWBG759 pDLK1	GQ900401.1 GU562624.1
**Pohoiki Harbor** **(** **station #33)**	Puna	anchialine pool	12/16/2020	89	MRSA	8	8	*blaZ* (*rep*_19_) *erm*(C) (*rep*_10_) *lnu*(A) (*rep*_21_)	-	pWBG759 pKLD1 pKH21	GQ900401.1 GU562624.1 EU350088.1
**Richardson’s Beach Park** **(station #2)**	Hilo	beach water	11/14/2020	81	MSSA	72	8	-	*aur* (*rep*_7c_)	-	BX571857.1
**Honoli** **ʻi Beach Park (station #2)**	Hilo	sand	12/14/2020	106	MSSA	72	8	-	*aur* (*rep*_7c_)	-	BX571857.1
**Richardson’s Beach Park** **(station #2)**	Hilo	beach water	08/21/2020	79	MSSA	1181	8	*tet*(K) (*rep*_7a_)	-	pS035-1	AM990993.1
**Lehia Beach Park** **(station #1)**	Hilo	sand	12/14/2020	111	MSSA	5	5	*erm*(T) (*rep*_13_) *tet*(K) (*rep*_7a_)	-	pLNU9 -	AM399082.1 AB037671.1
**Kealoha Beach Park (** **station #8)**	Hilo	beach water	10/31/2020	35	MSSA	5	5	*blaZ* (*rep*_20_)	*sed* (*rep*_20_)	SAP074A	GQ900426.1
**Onekahakaha Beach Park** **(station #9)**	Hilo	beach water	08/21/2020	30	MSSA	5	5	*blaZ* (*rep*_16_)	*-*	pSAS	BX571858.1
**Wailuku River–Upper** **(station #19)**	Hilo	river water	10/31/2020	14	MSSA	5	5	*blaZ* (*rep*_20_)	*sej* (*rep*_20_)	SAP074A	GQ900426.1
**Richardson’s Beach Park** **(station #3)**	Hilo	beach water	10/31/2020	46	MSSA	5	5	*mph*(C) (*rep*_19_)	-	pSJH901	CP000704.1
**Pohoiki** **(station #34)**	Puna	anchialine pool	09/18/2020	54	MSSA	6	5	*blaZ* (*rep*_16_)	-	pSaa6159	CP002115.1
**Lehia Beach Park** **(station #1)**	Hilo	beach water	10/31/2020	50	MSSA	398	398	*erm*(T) (*rep*_13_)	-	pC194	V01277.1
**Lehia Beach Park** **(station #1)**	Hilo	beach water	10/31/2020	84	MSSA	398	398	*erm*(T) (*rep*_13_)	-	pC194	V01277.1
**Kailua Pier** **(station #28)**	N. Kona	beach water	10/17/2020	58	MSSA	398	398	*erm*(T) (*rep*_13_)	-	pC194	V01277.1
**Pohoiki Beach** **(station #35)**	Puna	anchialine pool	12/16/2020	55	MSSA	398	398	*erm*(T) (*rep*_13_)	-	pC194	V01277.1
**Richardson’s Beach Park** **(station #3)**	Hilo	beach water	08/21/2020	45	MSSA	15	15	*blaZ (rep*_16_)	-	pSaa6159	CP002115.1
**Wailuku** **River Estuary (** **station #17)**	Hilo	river water	12/14/2020	16	MSSA	508	45	*blaZ (rep*_US5_)	-	unnamed	NC_003265.1

^a^ Multilocus sequence type determined by the allelic profile of seven *S. aureus* housekeeping genes: *arc*, *aro*, *glp*, *gmk*, *pta*, *tpi*, and *ygi*. ^b^ Clonal Complex (CC).

## Data Availability

All genomic data related to this project are available via NCBI GenBank under project PRJNA721726.
